# Principles to guide integrative oncology and the development of an evidence base

**DOI:** 10.3747/co.v15i0.278

**Published:** 2008-08

**Authors:** A.M. Leis, L.C. Weeks, M.J. Verhoef

**Affiliations:** * Department of Community Health and Epidemiology, University of Saskatchewan, Saskatoon, SK.; † Department of Community Health Sciences, University of Calgary, Calgary, AB.

**Keywords:** Integrative oncology, evidence-based medicine, randomized controlled trials, whole-systems research

## Abstract

**Background:**

Integrative oncology uses both conventional and complementary medicine to meet the needs of individual patients and to focus on the whole person. The core principles of integrative oncology include individualization, holism, dynamism, synergism, and collaboration, but the nature of the evidence to guide the development of integrative oncology has been given little attention.

**Objectives:**

- To discuss the need for evidence to support the integration of complementary therapies for integrative oncology care.
- To emphasize that the evidence base must be valid and respect the underlying principles of individual complementary therapies and integrative oncology practice.
- To suggest ways to begin developing the evidence base.

**Review and Discussion:**

Although the evidence for safety and efficacy seems paramount for supporting the integration of an individual complementary therapy into mainstream cancer care, the need for evidence to support the overall practice of integrative oncology has to be considered as well.

We argue that developing an evidence base for integrative oncology requires a contextual and comprehensive research approach that assesses a range of outcomes over a suitable period of time that the patient and the patient’s family, in addition to the health care providers, deem important.

**Conclusion:**

A whole-systems framework to the development of the evidence base for integrative oncology can guide the development of evidence that respects the complex nature of many complementary and integrative practices and their underlying principles of care delivery.

## 1. INTRODUCTION

The field of integrative oncology has emerged as a response both to cancer patients’ advocacy for holistic care and to an increasing evidence base for the safety and effectiveness of many complementary approaches, commonly called complementary and alternative medicine (cam). Cancer patients desire care that not only focuses on treating their disease, but also manages the course of their illness experience, optimizing health and enhancing well-being. Most cancer patients use complementary medicine alongside conventional medicine to meet these needs 1–4. Complementary and alternative medicine includes whole medical systems (Traditional Chinese Medicine, among others), mind–body medicine (for example, meditation), biologically-based practices (natural health products, for instance), manipulative and body-based therapies (for example, massage), and energy therapies (*qi gong,* among others) 5.

Integrative oncology uses both conventional medicine and cam to meet the needs of individual patients and focuses on the whole person 6. At the core of integrative oncology is the need for an evidence base to support the use of conventional and complementary treatments in a collaborative and synergistic manner. The nature of the evidence to guide the development of integrative oncology has, however, been given little attention. In the present paper, we discuss the need for evidence to support the integration of complementary therapies for integrative oncology care; we emphasize that the evidence base must be valid and must respect the underlying principles of individual complementary therapies and integrative oncology practice; and we suggest ways to begin developing that evidence base.

## 2. WHAT IS INTEGRATIVE ONCOLOGY?

The goal of integrative oncology is to support cancer patients and their families throughout the cancer journey by improving quality of life, ameliorating symptoms associated with conventional cancer care, alleviating distress, and in some cases, augmenting the effectiveness of conventional treatment 7,8. Mumber defines integrative oncology as “a comprehensive, evidence-based approach to cancer care that addresses all participants at all levels of their being and experience. It represents the next step in the evolution of cancer care in that it addresses the limitations of the current system while retaining the system’s successful features” 9.

Core principles of integrative oncology include individualization, holism, dynamism, synergism, and collaboration. In integrative oncology, the focus of care is on the whole person, and the aim is to promote the innate ability of each person to heal. Integrative oncology is individualized for each cancer patient over time, as each patient presents with unique symptoms and context, and as the goals of treatment change over time. Integrative care is also about compassion and caring for an individual in a holistic manner that gives voice to the patient’s values and needs. Grounded in a truly respectful partnership between patient and practitioner, a therapeutic alliance is forged that honours the patient’s informed choices. This collaborative approach to cancer care assumes that conventional and complementary practitioners—and patients—contribute their knowledge, experience, and skills to the healing encounter 8. In this context, a safe, knowledgeable, and dynamic cancer management plan is developed cooperatively, ensuring accurate monitoring and evaluation 10. Further, and in contrast to the prevailing pharmacologic model, the this cancer care approach recognizes the potential for synergy when therapies are integrated, with outcomes far exceeding the sum of the outcomes of individual therapies.

Integrative oncology is usually defined as an evidence-based discipline; however, we argue that the traditional (scientific) understanding of evidence needs to be revisited and expanded.

## 3. THE NEED FOR EVIDENCE

Integrative oncology makes a deliberate, yet fluid, distinction between complementary therapies, which are supported by evidence and used in combination with conventional cancer care, and alternative therapies, which are unproven and used as a replacement for conventional cancer care 7,11. When a strong evidence base is developed for some complementary therapies, they can potentially become part of integrative cancer care. For example, after a review of the available evidence, the Society for Integrative Oncology supports the use of acupuncture as a complementary therapy when cancer-related pain is poorly controlled 7.

The practice of integrative oncology therefore depends mainly on complementary therapies meeting standards of safety and, to a lesser extent, effectiveness (because the latter may be assessed from different perspectives in real-life situations). As a result, an understanding of what constitutes appropriate evidence is crucial to the foundation and future development of the field.

Despite agreement on the need for evidence to support the integration of complementary therapies into conventional cancer care 7,9,11, the discussion regarding the type of evidence required and its purpose is entirely based on evidence from specific cam treatments. We argue that this line of thinking misses an important point. To begin the discussion, a distinction must first be made between evidence for complementary therapies and evidence for integrative oncology practice. The first issue concerns evidence that supports the safety and effectiveness of individual complementary therapies, thus determining their suitability for integration into mainstream care. The second—and often forgotten—aspect concerns evidence that supports the synergistic integration of complementary and conventional practices in a collaborative and supportive manner within cancer care.

### 3.1 Nature of the Evidence Required for Complementary Therapies

According to Stark, Hess, and Shaw 12, different levels of evidence are required for the safety and effectiveness of individual complementary therapies depending on the goals of treatment. These levels of evidence depend on study design and sample size, and they range from well-designed randomized controlled trials [rcts (level 1)] to preclinical *in vitro* and *in vivo* studies and traditional medicines (level 4). Level 1 evidence is required for the use of complementary therapies with anti-neoplastic goals, but lower levels of evidence, such as nonrandomized trials or observational studies, are acceptable for less-invasive procedures and preventive or supportive goals. However, this hierarchy does not capture the multidisciplinary, synergistic approach that characterizes complementary therapies and integrative oncology alike in comparison with conventional care 10. Further, while addressing the need for evidence to support the individual integration of a complementary therapy into mainstream care, the need for evidence to support the overall practice of integrative oncology is often ignored.

A need arises to revisit traditional notions of evidence as they apply to complementary therapies. Traditional research methods are challenged in the attempt to evaluate complementary therapies, because these methods cannot account for the fundamental issues of individualization, synergism, and holism 8,9,13. These problems are compounded for the evaluation of integrative oncology, which involves the synergistic use of treatments from various healing paradigms and a range of physiologic, emotional, social, and spiritual outcomes.

### 3.2 Nature of the Evidence Required for Integration

Further to answering whether a complementary therapy works and is safe, questions regarding the appropriateness of integration must be examined. To this end, it is critical to document the ways in which complementary therapies and conventional care are being integrated and the outcomes that are important and relevant.

Integration can occur at many levels: individual, clinical, institutional, regulatory, or policy 14. Integration can also occur in many ways. For example, numerous patients are known to be integrating complementary therapies into their conventional care, but research is only starting to uncover how those patients make decisions regarding therapy selection, who is involved in the decision-making process, why the patients are integrating these therapies, and which outcomes are seen as relevant 15. Alternatively, health care providers may be the ones suggesting integration for their patients. The process of evaluation and decision-making is likely different in the two scenarios, in part because the intent may differ. For the health care professional, for example, the utmost concern is patient safety; but for patients, a decision to use cam may be driven by an attempt to minimize potential side effects and to feel empowered 16.

“Integrating” must also be distinguished from “combining.” “Combining” is more akin to adding various therapies to a treatment plan without considering the overall picture. “Integrating” involves synergistically applying a range of treatments to address holistic treatment goals as they change over time and in accordance with patient needs and values. Currently, although the goals and philosophy underlying integrative oncology are well developed, practical knowledge is not available concerning the extent to which and the manner in which diverse therapies are being integrated or combined.

Finally, integrative care seems to represent untapped opportunities (which so far remain understudied) for meeting the needs of cancer patients across the cancer trajectory.

## 4. VALIDITY AS CRITERION FOR EVIDENCE

As development of an evidence base begins both for individual complementary therapies and for integrative oncology, assurance is needed that the evidence base is valid—“validity” referring to the extent that appropriate research methods were used to support a conclusion regarding the efficacy or effectiveness of a therapy.

“Validity” commonly includes internal validity and external validity. Model validity is separate from both of those, but is just as important. Model validity is often overlooked because of the biomedical focus of most health care research. It refers to the extent to which the research methods used have addressed the unique theory and therapeutic context of the intervention being assessed 17.

Traditional clinical research methods have been developed to assess biomedical interventions, and thus model validity can typically be assumed. For research results to be valid in the case of integrative oncology, the research methods used must address the underlying principles of integrative oncology, such as its individualized, synergistic, holistic, and collaborative nature. The same is true for research regarding individual complementary therapies that are often based on assumptions contrary to biomedicine, such as the network of channels and blood vessels connected by *qi* (an essential fast-flowing substance full of vigour) in an approach using Traditional Chinese Medicine.

### 4.1 Limitations of the RCT Design for Complementary Therapies and Integrative Oncology

The double-blind rct is often upheld as the “gold standard” in clinical research, because of its strong internal validity arising from the ability to control for expected and unexpected bias, confounding factors, and error. However, it is impossible to achieve model validity while applying the blinded rct design to the practice of integrative oncology and many complementary therapies 13.

Sagar 8 highlights several of the key challenges in applying the rct design to the study of select complementary therapies. Examples include difficulties in determining appropriate placebos or sham treatments, the impossibility of double-blinding when the practitioner is part of the intervention, and problems respecting the individualized approach of many complementary practices. Acupuncture provides a good example, because the choice of an appropriate sham treatment for acupuncture has been an ongoing challenge 18, not unlike that in determining an appropriate sham treatment for surgery in the realm of conventional care. Further, blinding patients and providers is difficult, because both are quite aware of whether needling has taken place, although single-blinding may be possible if simulated needling is used as a sham treatment. In practice, different needling protocols are developed for specific patients, depending on their unique symptoms and holistic context, and thus standardization of an acupuncture protocol for a rct is problematic if model validity is to be upheld.

The same argument can easily be extended to integrative oncology practice, in which therapies from diverse philosophical backgrounds are combined, thus making model validity even more difficult to attain. In addition to the problems of defining a placebo, blinding, and standardization, the rct is not designed to measure the effect that each patient’s unique physical, social, and cultural context and corresponding reasons for integration may have on treatment outcomes. Further, the rct cannot assess the synergism that results from the integration of various therapies, coupled with the healing context and the clinical skills and expertise of the integrative team.

### 4.2 Building Evidence for Integrative Oncology

Developing an evidence base for integrative oncology requires a contextual and comprehensive research approach that assesses a range of outcomes over a suitable period of time that the patient, the patient’s family, and health care providers deem important. We next highlight some approaches that begin to meet the requirements of internal, external, and model validity. Readers should consult the papers referenced in this section for detailed descriptions of these approaches.

#### 4.2.1 Variations of the RCT Design

Although the rct in its classical form cannot meet the requirements for model validity when applied to complementary therapies or integrative oncology, some variations have been suggested that address many of the shortcomings. For example, pragmatic rcts do not require standardization of the intervention and thus allow for the individualized nature of the treatments to be assessed. Preference rcts account for the effect that beliefs and preferences of patients for certain treatment types will have on treatment outcomes. In a preference rct, patients with treatment preferences receive their preferred treatment; patients who do not have a preference are randomized as usual.

#### 4.2.2 The Power of Mixed Methods

Although quantitative research approaches have been well established in evaluating treatment interventions, the importance of qualitative research cannot be overlooked or dismissed as development begins of the evidence base for complementary therapies and integrative oncology. Qualitative research has amazing potential to explore, in depth, from a variety of perspectives (patients, oncology team, cancer care system), how integrative cancer treatment and care are experienced. Including qualitative inquiry in the evaluation of interventions should be an integral part of evidence-based medicine 19.

Qualitative research aims to understand the nature of phenomena; it will prove crucial as exploration of the potential of integrative oncology for cancer care moves forward. For example, qualitative research is ideally suited to answer the question of what integrative oncology is, how it is being practiced, how it can best be practiced, and the benefits that are possible. Such exploratory work can help to elucidate the key components in integrative care and their synergistic relationships from the viewpoint of patients and of practitioners.

Because qualitative research can fill an important niche in this field, it should, when possible, be nested within clinical trials or other quantitative designs 20,21 as a form of mixed-methods research. Such a design represents a further modification of the rct method that addresses the requirements of internal, external, and model validity. This approach can help to elicit whether, why, and how patients benefit from a complex intervention and can explore relevant outcomes from a variety of perspectives.

#### 4.2.3 Whole-Systems Research

Whichever research methods are ultimately adopted to study complementary therapies and integrative oncology, investigators must systematically capture the complexity inherent in these approaches to healing. A “whole-systems” research framework is helpful to conceptualize the important issues that need attention and to design step-wise programs of research that will answer the multifaceted questions.

The notion of whole-systems research has recently been given attention in the literature, both with regard to complementary therapies 13,22 and integrative oncology 23,24. In a general sense, the goal of whole-systems research “is to appropriately combine research designs and methods in a coherent research program, so that all aspects of an internally consistent approach to treatment, or a whole system, can be assessed. It acknowledges an individualized, patient centered and participatory approach to diagnosis and treatment and a process of healing that collaboratively combines patient and practitioner knowledge and skills, thus enhancing healing” 23.

The relevance of a whole-systems framework to the development of the evidence base for integrative oncology should be apparent. It can guide the development of evidence that respects the complex nature of many complementary and integrative practices and their underlying principles of care delivery 25. A program of research guided by a whole-systems research framework would include rcts, variations of rcts, observational trials, and qualitative research, ideally mixing methods as appropriate, depending on the specific focus. A program of whole-systems research would thus produce research that collectively has strong validity.

## 5. CONCLUSIONS

As cancer patients increasingly turn to cam as a way to complement their cancer care, it is crucial that health care professionals become informed about the evidence base behind this group of practices 26 and, further, that they remain critical of the need for valid research. The assessment of validity for complementary therapies and integrative oncology alike should encompass internal, external, and model validity. Because it seems unlikely that any one study design can achieve optimal levels of each type of validity, health professionals and researchers need to be open to emerging models of evidence that are not necessarily aligned to traditional ideas of evidence in biomedicine. Programs of research have to include a variety of evidence types and treat all of them as legitimate. A whole-systems research framework is helpful to guide the development of these research programs.
